# A comparison of stability of chemical analytes in plasma from the BD Vacutainer^®^ Barricor™ tube with mechanical separator versus tubes containing gel separator

**DOI:** 10.1002/jcla.23060

**Published:** 2019-10-11

**Authors:** Ghassaan Gawria, Linda Tillmar, Eva Landberg

**Affiliations:** ^1^ Department of Clinical Chemistry and Department of Clinical and Experimental Medicine Linköping University Linköping Sweden

**Keywords:** phosphate, plasma, potassium, separator, stability, tube

## Abstract

**Background:**

There is a need of prolonged stability of certain chemical analytes in lithium heparin tubes with separators. A new tube with a mechanical separator has recently been launched (Barricor™), which according to the manufacturer may have these benefits. The aim of this study was to evaluate stability performance of this tube in comparison with plasma gel tubes under clinically realistic circumstances.

**Methods:**

Blood was collected in tubes containing lithium heparin with different separators; gel separator (Vacutainer^®^ PST™, Becton Dickinson and Vacuette^®^, Greiner bio‐one) and mechanical separator (Vacutainer^®^ Barricor™, Becton Dickinson). All tubes had an aspiration volume of 3 mL and were centrifuged at similar time and force. Tubes were transported manually or by car. Seven analytes from 122 patients were analyzed after 3 to 80 hours by Cobas c701 (Roche).

**Results:**

The Barricor™ tube showed increased stability of phosphate and potassium and similar stability of aspartate aminotransferase, glucose, homocysteine, lactate dehydrogenase, and magnesium compared with gel tubes. Maximal allowable bias for phosphate was exceeded after 68 hours for Barricor™ tubes compared with 29 or 35 hours for gel tubes and for potassium after 40 hours for Barricor™ tubes vs 9 or 12 hours for gel tubes. Transportation did not affect stability. Hemolysis index was slightly lower in Barricor tubes than in gel tubes (*P* = .01).

**Conclusion:**

Implementing the new Barricor™ tube will improve stability of potassium and phosphate in plasma. Blood sampling facilities far from the laboratory may benefit from using these tubes, thus diminishing preanalytical errors.

## 1 INTRODUCTION

The introduction of gel separator tubes for venous blood collection has revolutionized handling of tubes at laboratories. The increased stability of several analytes in gel tubes diminishes the need of transfer to and relabeling of a secondary tube. The latter procedure will otherwise lead to increased risk of patient identification mix‐up, which may lead to serious clinical consequences. However, especially for plasma gel tubes, the stability of certain analytes is diminished compared with plasma transferred to a secondary tube before storage or transportation. This is particularly true for potassium, where several studies have shown falsely elevated levels after storage more than 12‐20 hours or after transport.^1^, ^2^, ^3^ Other analytes often measured in routine clinical chemistry that show reduced stability in gel tubes are aspartate aminotransferase (AST), glucose, lactate dehydrogenase (LDH), and phosphate.^2^ The explanation to these findings is most likely that plasma is not free of cells. Evaluations by tube manufacturers have shown that a small proportion of platelets and lymphocytes exists on the gel surface and also sometimes as aggregates in the top layer.^4^ Some erythrocytes may also remain in the plasma after centrifugation.^5^ The increase in potassium and other components could thus be explained by leakage from cells, with the highest impact on results when intracellular content is higher than in plasma.

**Figure 1:**
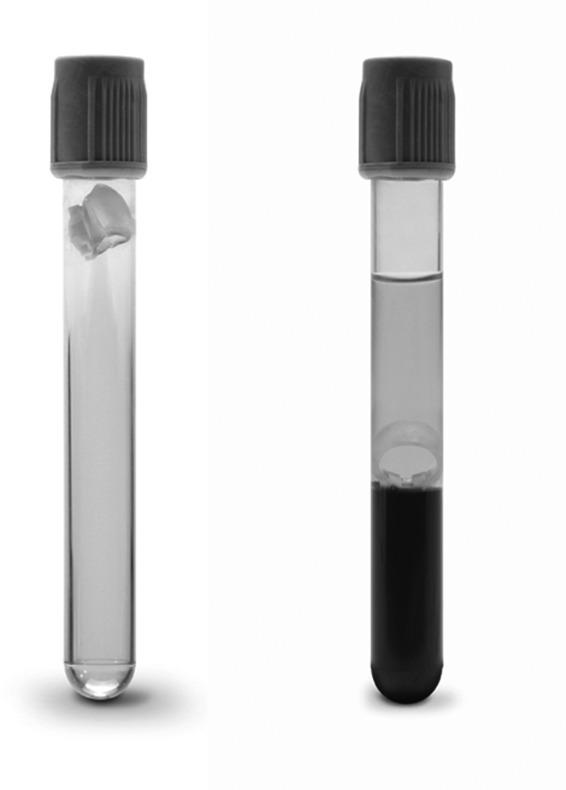
Tubes with mechanical separator (Vacutainer^®^ Barricor™). The figure shows one unused tube and one tube after blood collection and centrifugation. Courtesy and ^©^Becton, Dickinson and Company. Reprinted with permission

A tube with a new type of mechanical separator was recently launched by Becton Dickinson (BD). The separator consists of a flexible elastomer part and a robust plastic part, which ensures right positioning of the separator during centrifugation (Figure 1). This type of separator should lead to less hydrophobic interaction with analytes that previously was affected by the gel.^6^ There is also hope for increased stability of analytes after centrifugation based on a better separation of plasma from blood cells. Until today, there is only limited independent knowledge concerning stability of chemical analytes in BD Barricor™ tubes. In one study, stability of potassium in these tubes was examined for a short storage period of 15‐hours 7 and 10‐day stability of five analytes has also been studied in samples from only 4 individuals.^8^ A study by Demeester et al examined stability of 21 routine chemistry tests in BD Barricor™ tubes for up to 7 days.^9^ However, this study included only healthy volunteers and used different centrifugation settings and a tube without gel for comparison. The aim of this study was to measure stability of 7 common chemical analytes expected to be sensitive to prolonged storage in the primary tube and compare stability in the Vaccutainer^®^ Barricor™ tube with similar tubes containing gel separator for up to 80 hours. To make this comparison independent of other factors that may affect stability, we designed the study so that sampling technique, aspiration volume, and centrifugation conditions would be the same for all type of tubes. We used samples from patients and divided the study into two parts with different tube transportation procedures to simulate the preanalytical workflow in reality.

## 2 MATERIAL AND METHODS

### 2.1 Selection of patients

In part one of the study, patients admitted to the hospital‐based unit for blood sampling at Linköping University Hospital were asked to participate in the study. A total of 20 patients (8 women and 12 men) with a median age of 50 years (25‐69) were included. Seven patients had decreased kidney function with an estimated glomerular filtration rate (eGFR) below 60 mL/min/1.73 m^2^; two of them had eGFR less than 20 mL/min/1.73 m^2^. Three patients had diabetes and one patient was on anticoagulant therapy (Tinzarapin). Three patients had slight leukocytosis (>9.0 × 10^9^/L) and two patients had slight thrombocytosis (>350 × 10^9^/L).

In part two of the study, patients admitted for blood sampling at two outpatient primary care units in the province of Östergötland were asked to participate in the study. Unit A was situated at a distance of 20 km and unit B at a distance of 54 km from the hospital laboratory, measured by transport route. A total of 102 patients, 51 patients from each unit, were included. Among the patients, there were 42 women and 60 men, with a median age of 61 years (21‐86). Out of these, 15 patients had a known decreased kidney function, 14 had diabetes, and 3 patients had both a decreased kidney function and diabetes (based on eGFR <60 mL/min/1.73 m^2^ or HbA1c above age‐dependent reference range).

### 2.2 Blood sampling and handling

Blood sampling was performed by experienced laboratory staff, and blood was collected by venipuncture in the antecubital vein. The procedure for blood sampling followed the EFLM recommendation on venous blood sampling.^10^

In part one, blood was collected in two different vacuum tubes both containing lithium heparin as anticoagulant and with a 3‐mL aspiration volume. One of the tubes contained gel as separator (Vacutainer^®^ PST™, BD), and the other new type of tube contained a mechanical separator consisting of a flexible elastomer part connected to a firm plastic part (Vacutainer^®^ Barricor™, BD, Figure 1). The order of sampling was varied systematically. As reference, a plasma tube with gel (Vacutainer^®^ PST™ LH, 3 mL, BD) was used and always drawn before the other two tubes. After 15‐30 minutes, centrifugation was performed in a swinging bucket centrifuge at 2400 g for 5 minutes at RT (20°C). This centrifugation mode was in agreement with the local routine at the laboratory but was not according to present recommendations by BD. On inspection, all separators were situated at the ordinary position above the cell layer. Plasma in the reference tube was immediately and carefully transferred by a pipette to a secondary tube. Centrifuged tubes awaiting analysis were stored at 4°C. Transportation of tubes was performed manually in an upright position.

In part two, blood was collected in three different vacuum tubes, all containing lithium heparin as anticoagulant and with a 3 mL aspiration volume: Vacutainer^®^ PST™ gel separator (BD), Vacutainer^®^ Barricor™ (BD), and Vacuette^®^ gel separator (Greiner bio‐one). Centrifugation was performed at 2400 g for 10 minutes at RT, in agreement with recommendations from BD and Greiner bio‐one. Vacutainer^®^ PST™, LH, 3 mL (BD) was used as reference. As in part one, the order of sampling was varied and plasma in the reference tube was immediately and carefully transferred by a pipette to a secondary tube. All tubes were stored at 4°C awaiting transport and analysis. Tubes were transported to the hospital laboratory by car in an upright position. Temperature during transport was not measured, but was conducted according to normal routine; that is tubes were transported mainly at RT. Part two was performed in March to April 2018.

### 2.3 Analyses

Analysis was performed on Cobas c701 (Roche Diagnostics) with reagents and calibrators from Roche. Quality controls on two levels were run regularly 2‐3 times per day, and patient sample results were only reported if these measurements reached specified quality goals. In part one, aspartate aminotransferase (AST), glucose, homocysteine, lactate dehydrogenase (LDH), magnesium, phosphate, potassium, and hemolysis index (HI) were measured instantly after centrifugation (time point 0) and then henceforth 6, 24, 48, and 72 hours after the first measurement. The tubes were recapped shortly after analysis. All tubes were stored at 4°C awaiting analysis. In part two, all tubes collected from each patient were analyzed simultaneously and the same parameters as in the study part one were measured. Analysis was performed shortly after arrival at the hospital laboratory. HI of 100 units is equivalent to 1 g/L hemoglobin according to Roche and our own evaluation.

### 2.4 Ethical considerations

The study was approved by the Regional Ethics Committee at Linköping University Hospital (2016/437‐31 and 2017/587‐32) and performed in accordance with the Declaration of Helsinki. All participants were informed about the purpose and procedure of the study and gave their informed consent.

### 2.5 Statistics

All data were checked for normal distribution by the test of Kolmogorov‐Smirnov. The difference in percentage from the reference tube at each time point was calculated for each analyte and tube by the following equation.

$$\text{difference}\;\left(\%\right)=\frac{\text{result}-\text{result}\;\text{reference}\;\text{tube}}{\text{result}\;\text{reference}\;\text{tube}}\times 100$$

**Figure 2:**
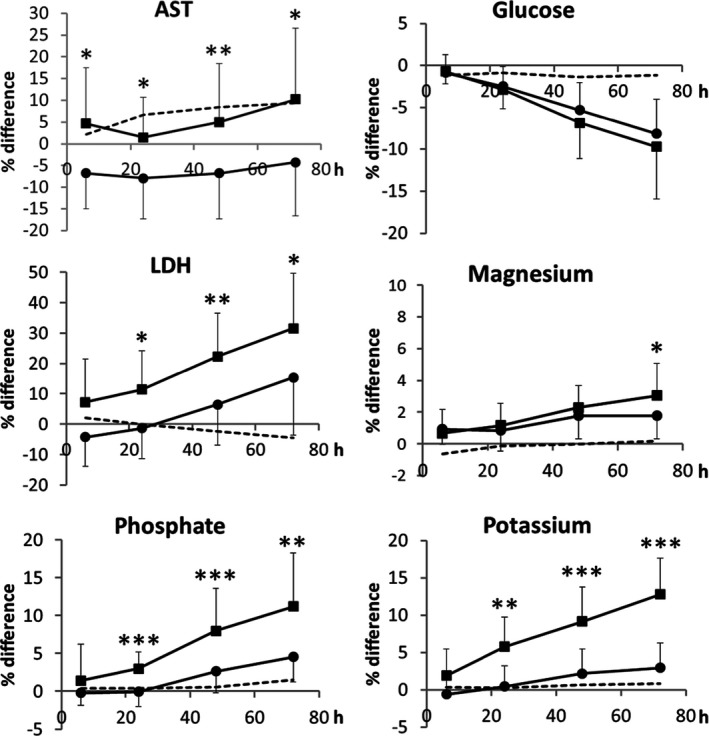
Influence of storage time at 4°C for six analytes in two different types of lithium heparin plasma tubes. Squares correspond to BD gel tube, circles to BD Barricor™ tube, and the striped line “‐‐‐‐“ to reference tube. Plasma in the reference tube was transferred to a secondary tube after the first measurement. The difference at each time point was calculated as percentage of the mean deviation from the measurement in the reference tube at the same time point. Difference showed for the reference tube is in relative to the measurement at 0 h. Bars represent SD. Stars mark the level of significance between data measured in Barricor™ tubes compared with gel tubes at each time point. According to Bonferroni correction, the stars indicate following levels of *P*‐values; *P < .0125, **P < .0025, and ***P < .00025

In part one, the difference (%) for all parameters and time points were normally distributed. Thus, mean and SD was calculated (Figure 2). Significance of differences between the two tube types was examined for each parameter and time point by 2‐sided paired Student´s t‐test. Bonferronis correction was used to adopt the level of significant p‐value to four measurements (four time points). Thus, a *P*‐value <.0125 was regarded as a significant difference. As the analyte concentrations did not follow a normal distribution, Friedmans ANOVA was used to check for significant change from 0 to 48 hours (Table 1). Data from the measurements at 72 hours were excluded due to two missed samples.

**Table 1:** Influence of storage at 4°C on the analyte concentration in two tubes from BD with different type of separators

Analyte (unit)	Type of tube	Median (range) 0 h	Median 6 h	Median 24 h	Median 48 h	Change *P*‐value
AST (µkat/L)	Barricor	0.35 (0.20‐0.73)	0.35	0.37	0.36	.027
	Gel, PST	0.39 (0.22‐0.92)	0.39	0.41	0.40	.019
	Ref, PST	0.40 (0.23‐0.87)	0.38	0.38	0.38	.051
Glucose (mM)	Barricor	5.44 (3.74‐11.8)	5.40	5.36	5.20	<.001
	Gel, PST	5.46 (3.79‐11.8)	5.42	5.35	5.19	<.001
	Ref, PST	5.51 (4.03‐11.9)	5.45	5.46	5.42	<.001
Homocysteine (mM)	Barricor	14.4 (6.0‐147)	14.1	14.6	14.0	.70
	Gel, PST	14.2 (6.5‐148)	14.2	14.5	13.9	.31
	Ref, PST	14.4 (6.3‐150)	14.2	14.3	13.5	.046
LDH (µkat/L)	Barricor	2.8 (1.6‐4.8)	2.8	2.9	3.0	<.001
	Gel, PST	3.0 (1.8‐6.0)	3.2	3.2	3.5	<.001
	Ref, PST	3.0 (2.0‐5.5)	3.0	2.9	2.8	<.001
Magnesium (mM)	Barricor	0.76 (0.57‐0.88)	0.76	0.76	0.77	<.001
	Gel, PST	0.76 (0.57‐0.88)	0.76	0.75	0.77	<.001
	Ref, PST	0.77 (0.58‐0.88)	0.76	0.78	0.79	.50
Phosphate (mM)	Barricor	0.98 (0.63‐1.25)	0.95	0.97	1.00	<.001
	Gel, PST	0.97 (0.63‐1.27)	0.97	0.99	1.04	<.001
	Ref, PST	0.97 (0.64‐1.27)	0.96	0.96	0.97	.54
Potassium (mM)	Barricor	4.00 (3.34‐4.45)	4.06	4.11	4.21	<.001
	Gel, PST	4.09 (3.39‐4.70)	4.18	4.30	4.44	<.001
	Ref, PST	4.03 (3.36‐4.56)	4.10	4.07	4.08	.17

In part two, correlation between results and time to analysis was examined using Spearman rank order correlation (Table 2). Time to exceed allowable bias was calculated using a linear regression model (Table 2). To examine the significance of difference for hemolysis between tubes, Wilcoxon matched pair test was used. Statistic software used was Excel 2010 and Statistica 13.

**Table 2:** Correlation between bias and time to analysis in three different lithium heparin tubes with separators

Analyte	Type of tube^a^	Slope (95% CI)	Intercept (95% CI)	MAB^b^ (%)	Time to MAB^b^	Rs (*P*‐value)
AST	Barricor BD	−0.069 (−0.145 to 0.007)	5.3 (2.1‐8.5)	5.4	80 h	−0.17 (.086)
	Gel BD	−0.041 (−0.116 to 0.034)	7.5 (4.3‐10.7)		0 h^c^	−0.065 (.53)
	Gel Greiner	−0.055 (−0.135 to 0.026)	8.2 (4.8‐11.6)		0 h^c^	−0.080 (.43)
Glucose	Barricor BD	−0.106 (−0.136 to −0.077)	−0.57 (−1.8‐0.68)	2.5	18 h	−0.63 (<.001)
	Gel BD	−0.100 (−0.125 to −0.076)	0.30 (−0.74‐1.33)		28 h	−0.68 (<.001)
	Gel Greiner	‐0.107 (−0.128 to −0.086)	0.29 (−0.58‐1.2)		26 h	−0.76 (<.001)
Homocysteine	Barricor BD	0.004 (−0.010 to 0.019)	1.6 (0.96‐2.2)	7.7	>80 h	0.036 (.73)
	Gel BD	0.003 (−0.019 to 0.013)	1.6 (0.48‐1.8)		>80 h	0.054 (.60)
	Gel Greiner	0.013 (−0.004 to 0.031)	0.84 (0.11‐1.6)		>80 h	0.15 (.14)
LDH	Barricor BD	0.170 (0.094 to 0.24)	15 (11‐18)	4.3	0 h^c^	0.41 (<.001)
	Gel BD	0.225 (0.144 to 0.306)	16 (13‐19)		0 h^c^	0.52 (<.001)
	Gel Greiner	0.174 (0.095 to 0.254)	17 (13‐20)		0 h^c^	0.44 (<.001)
Magnesium	Barricor BD	0.004 (−0.006 to 0.014)	1.5 (1.1‐1.9)	1.8	75 h	−0.023 (.82)
	Gel BD	0.006 (−0.007 to 0.019)	1.0 (0.47‐1.5)		>80 h	0.077 (.45)
	Gel Greiner	0.036 (0.010 to 0.062)	4.0 (2.9‐5.1)		0 h^c^	0.13 (.20)
Phosphate	Barricor BD	0.036 (0.024 to 0.047)	0.77 (0.27‐1.3)	3.2	68 h	0.50 (<.001)
	Gel BD	0.070 (0.056 to 0.084)	1.2 (0.64‐1.8)		29 h	0.70 (<.001)
	Gel Greiner	0.060 (0.047 to 0.072)	1.1 (0.59‐1.7)		35 h	0.71 (<.001)
Potassium	Barricor BD	0.036 (0.024 to 0.048)	0.37 (−0.14‐0.88)	1.8	40 h	0.53 (<.001)
	Gel BD	0.094 (0.081 to 0.107)	0.98 (0.42‐1.5)		8.7 h	0.82 (<.001)
	Gel Greiner	0.101 (0.084 to 0.118)	0.60 (−0.11‐1.3)		12 h	0.77 (<.001)

a Barricor BD refers to Vacutainer^®^ Barricor™ LH 3 mL (BD), Gel BD to Vacutainer^®^ PST™ LH 3 mL (BD) and Gel Greiner to Vacuette^®^ LH 3 mL Sep (Greiner bio‐one).

b MAB; maximal allowable bias, according to Ricos et al.^11^

c Initial bias exceeds the maximal allowed bias when compared to aliquoted plasma from BD's PST™ Gel tube (reference tube).

## 3 RESULTS

The influence of storage time on 7 especially sensitive analytes was compared between two types of plasma lithium heparin tubes, containing different cell separators (BD PST™ and BD Barricor™). Samples from twenty patients were collected by venipuncture and analyzed after storage at 4°C for 6, 24, 48, and 72 hours. Two samples failed to be measured after 72 hours, and magnesium was only measured in samples from 18 subjects.

All analytes except homocysteine changed significantly during storage for up to 48 hours (Table 1). AST, LDH, magnesium, phosphate, and potassium increased with storage time and glucose decreased (Figure 2). There was also a near significant increase of AST in the reference tube (*P* = .051) during storage. Figure 2 shows the change relative to the reference tube at each time point. Significant differences between the BD gel and BD Barricor tube were found for AST already at time point 6 hours, for LDH, phosphate, and potassium from time point 24 hours and for magnesium first at time point 72 hours.

**Figure 3:**
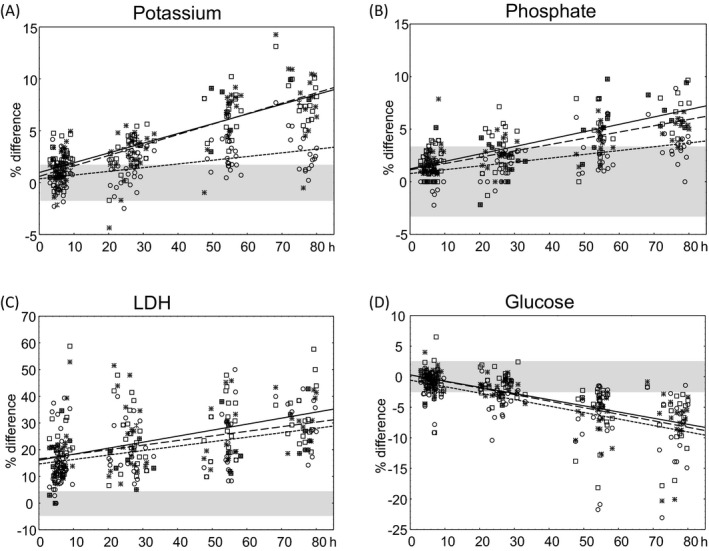
Influence of transport and storage (at 4°C) on A, potassium, B, phosphate, C, LDH and D, and glucose measured in three different types of lithium heparin plasma tubes. Difference in percentage as compared to the reference tube is plotted against time to analysis. Squares and the continuous line correspond to BD gel tubes, stars and the line with long stripes correspond to Greiner bio‐one gel tubes, whereas circles and the line with short stripes correspond to BD Barricor™ tube. Gray areas indicate limits of maximal allowable bias

In part two, samples were collected from 102 patients at two different outpatient wards. The aim of this part of the study was to evaluate the BD Barricor™ tube during more realistic circumstances including the transport of tubes to the laboratory. Plasma from all tubes was analyzed concurrently at one time point per patient. Bias was calculated in comparison with the reference tube (Figure 3). Samples from one subject were excluded because of hemolysis (>0.5 g/L). Only LDH, phosphate, and potassium showed a significant positive correlation with storage time, whereas glucose showed a significant negative correlation with storage time (Table 2, Figure 3). Significant differences between BD Barricor™ and gel tubes (both providers) were found for phosphate and potassium, with lower bias for the BD Barricor™ tubes (Figure 3A and 3). The maximal allowable bias for potassium, according to Ricos et al,^11^ was exceeded after 40 hours for BD Barricor™ tubes, but already at 9 or 12 hours for BD gel tubes and Greiner bio‐one gel tubes, respectively (Figure 3A). As for phosphate, the maximal allowable bias was exceeded after 68 hours for BD Barricor™ tubes and at 29 or 35 hours for gel tubes (Figure 3B). Also in part two of the study, there was a considerable bias for LDH already at the early measuring points (3‐10 hours). As seen in Figure 3C, the bias was approximately 15% for all tube types, which was higher than in the first part of the study (Figure 2) and far to exceed the allowable bias of 4.3%.^11^ Also AST showed an initial increase in all tubes, which exceeded the maximum allowable bias in gel tubes. Since AST also increased in the aliquoted plasma in the reference tube, according to part one, the calculated difference compared with the reference tube probably masks the true change during storage. Glucose decreased during storage in all three tubes. No significant difference was found between glucose measured in BD Barricor™ compared with gel tubes (Figure 3D).

Except for magnesium, there was no significant difference between analytes measured in gel tubes from BD compared with gel tubes from Greiner bio‐one at any time point. Magnesium concentration was on average 0.03 mmol/L higher in gel tubes from Greiner bio‐one (*P* < .001), independent of time to analysis.

In part two, the increase of potassium and phosphate in gel tubes during storage was smaller than in part one. However, stability of potassium, phosphate, glucose, and LDH was similar in Barricor tubes in both parts of the study, indicating no substantial impact of centrifugation time or the rough conditions during transportation (Figures 2 and 3). To further evaluate the effect of transportation, the increase of potassium in BD Barricor™ tubes after 47‐80 hours was chosen and the variation was compared between the two study parts. In part one, the SD of % difference at 48 and 72 hours was 1.7 and 1.9%, respectively. In part two, the SD of % difference at 47‐80 hours (n = 37) was 1.8%.

**Figure 4:**
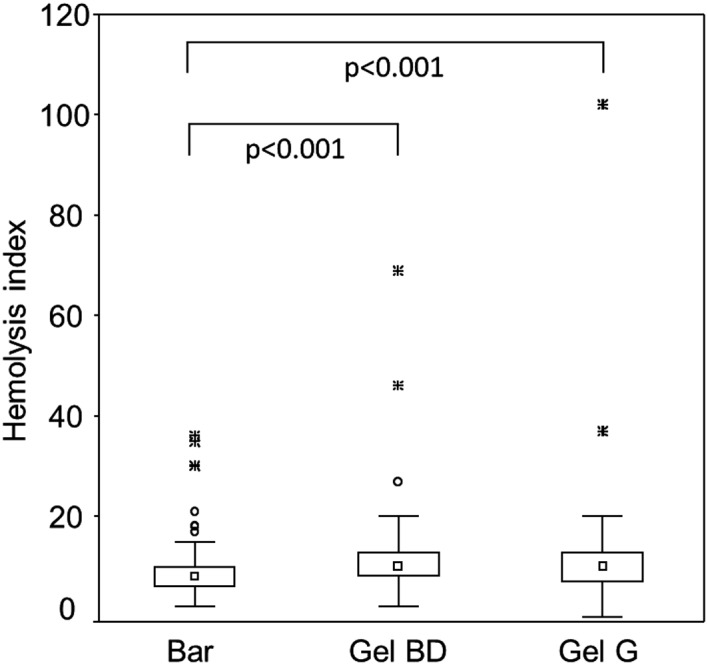
Hemolysis index measured in three types of lithium heparin plasma tubes collected from 102 patients in primary care. Blood samples were collected by venipuncture in tubes with a mechanical separator from BD (Bar), in tubes with a gel separator from BD (Gel BD) and in tubes with a gel separator from Greiner bio‐one (Gel G). The order of sampling was varied systematically. HI of 100 is equivalent to 1 g/L hemoglobin. The point label indicates the median. The box indicates the 25th to the 75th percentiles and the bars indicate non‐outlier range. Circles mark outliers and stars mark extremes as defined by a deviation of >1.5 or >3 times the interval between the upper and lower value of the box

Hemolysis index (HI) was measured in all samples (n = 122) at the first time point. HI was generally low, and clearly visible hemolysis (above 0.5 g/L) was only found in 3 out of 122 BD gel tubes and in none of the BD Barricor™ tubes. Statistically, a small but significant difference (*P* < .001) was found between BD Barricor™ tubes and both gel tubes, with a tendency of lower HI in BD Barricor™ tubes (Figure 4).

## 4 DISCUSSION

A large number of blood samples are collected in primary care and other outpatient wards. Transport to the central laboratory is seldom immediate and often delayed to the next day, sometimes several days. It is therefore a great advantage if prolonged stability for certain parameters in plasma tubes can be achieved, thus avoiding the need for plasma transfer to a secondary tube.

By using the newly introduced tube with mechanical separator (Barricor™, BD), there is a potential to achieve better stability of certain chemical analytes where cellular leakage may be a problem. Recently, 77 routine clinical chemistry analytes were evaluated in plasma from Barricor™ tubes and compared with results from gel tubes.^12^ Except for LDH, no significant difference was found between tubes centrifuged at similar force and time (2000 g, 10 minutes). However, in this previous study, analysis was done immediately after centrifugation and stability during storage was not addressed. LDH was found to be higher in gel tubes compared with Barricor™ tubes, which is in accordance with our study at the hospital unit. This difference was not as clear in the part two of our study, where measurements in both tubes showed increased values already at an early time point (<10 hours), compared with the reference tube. It is difficult to explain these findings, but it should be emphasized that plasma is probably not a reliable system for measurement of LDH. Centrifugation conditions may also affect the level of LDH as seen in previous studies where a higher level of LDH was found after increasing the centrifugal force.^13^, ^14^ This may explain the discrepancy of the bias of LDH between samples centrifuged for 5 and 10 minutes respectively, found in our study.

In this study, we found that the stability of AST, glucose, LDH, and potassium in lithium heparin gel tubes was similar to previous findings.^2^, ^7^ Phosphate and potassium were clearly more stable in the mechanical separator tubes (Barricor™) than in the gel tubes.

Compared with gel tubes, potassium in Barricor™ tubes showed increased stability up to 80 hours and reached maximum allowable bias after 40 hours compared with 9 or 12 hours for gel tubes. The increased stability in Barricor™ tubes is in accordance with a study were stability of potassium was tested up to 15 hours.^7^ The allowable bias of 1.8% for potassium based on Ricos et al^11^ may be questioned, as this only represent 8% of the reference range for plasma potassium (3.5‐4.4 mmol/L). Some authors have used a higher maximum bias (2.8% and 4.0%, respectively) as a quality goal.^7^, ^15^ Though, even the higher goal of 4.0% is exceeded after 32 or 34 hours in gel tubes from BD or Greiner bio‐one, whereas potassium in Barricor™ tubes reaches this bias after >80 hours.

Phosphate also showed increased stability in Barricor™ tubes during storage. Potassium and phosphate are both electrolytes with considerably higher concentration intracellular than extracellular. Additionally, they are well regulated in plasma, reflected by narrow reference intervals and high clinical demands on analytical accuracy.

Hemolysis as measured by hemolysis index on Cobas c701 was generally low in all tubes, but statistically significant lower values were found for Barricor™ tubes. Hemolysis is clearly visible at approximately 0.5 g hemoglobin per liter, which corresponds to a HI of 50. This level of hemolysis may have clinical implications for analytes such as potassium, AST, and LDH. Clinical significant hemolysis above this level was not found in any of the Barricor™ tubes, whereas 3 out of 122 samples collected in BD gel tubes showed hemolysis at this level. This is an interesting finding, but needs to be confirmed by studies including a larger amount of samples, preferentially collected in different clinical situations. Risk of hemolysis may also depend on the tube vacuum, and partial draw blood collection tubes are beneficial.^16^, ^17^ In this study, we therefore compared tubes with similar draw volume (3 mL) and also used tubes with partial draw volume.

Samples were always handled in a vertical position during storage, package, and transport. This procedure is important to avoid preanalytical error.^18^ That also means that our findings are only valid under these conditions.

Transportation under rough conditions, as in part two, did not show any effect on the stability of the measured parameters in Barricor™ tubes. On the contrary, there was a tendency toward a lower bias for potassium and phosphate in gel tubes, which may be related to the longer centrifugation time in part two. Increasing centrifugation time and force may result in lesser amount of cells trapped in plasma and thus lead to increased stability over time.^19^ However, for Barricor™ tubes, centrifugation at 2500 g 5 minutes gave similar results compared with 2500 g 10 minutes and LDH even showed less bias against aliquoted plasma when centrifugation was performed at 5 minutes. This shows the importance of optimizing centrifugation conditions for each tube and analysis. In addition, temperature during centrifugation and storage may also affect stability and have to be considered when setting up routines for sample handling.

When measuring plasma glucose, there is a need of avoiding decreasing values caused by glycolysis. The recommendation is therefore to use special tubes with citrate, EDTA, and sodium fluoride. These additives will stop the glycolysis immediately. If time to centrifugation cannot be kept <10 minutes, tubes with these kinds of glycolytic inhibitors should be used.^20^ However, if this preanalytical step is well controlled, our study shows that glucose can be measured up to approximately 20 hours in Barricor™ or gel tubes stored at 4°C without exceeding the allowable bias of 2.5%. In contrast to potassium and phosphate, we could not find a significant difference between Barricor™ and gel tubes for glucose. This is perplexing, since the cellular content in plasma from Barricor™ tubes is lower according to BD and therefore theoretically should decrease the rate of glucose metabolism. An explanation to this finding may be that the cellular distribution also plays a part. Two studies have found that it is mainly erythrocytes and leukocytes that are diminished in plasma in Barricor™ tubes compared with gel tubes.^19^, ^21^ Platelet numbers were similar compared with gel tubes. A relatively higher glucose metabolism rate in platelets than in other blood cells may explain the lack of effect on glucose stability in Barricor™ tubes compared with gel tubes.

Most previous studies on stability of analytes in plasma have used blood collected from healthy individuals. This may overestimate the stability of some substances, especially compared to analytes measured in plasma from patients with severe diseases or deranged proportions of blood cells. Therefore, in this study we included patients with different acute or chronical diseases. Further studies are needed to evaluate stability in samples from patients with marked thrombocytosis, polycythemia, or leukocytosis, which theoretically may have an impact on number of cells trapped in plasma and thus the degree of stability of certain parameters.

One may argue that most of the analytes in this study are more stable in serum and that tubes with coagulation activators should be used when prolonged storage is needed. However, there are other disadvantages with serum compared with plasma.^22^ Plasma tubes may be centrifuged immediately, while blood in serum tubes must be allowed to rest and needs to be checked for sufficient coagulation. In serum, there will also be a platelet dependent bias of both potassium and phosphate.^23^

In conclusion, use of the new Barricor™ tube, instead of gel tubes, will improve stability of potassium and phosphate in plasma. Hemolysis was also significantly lower in these tubes, but further studies are needed to evaluate the clinical impact of this finding. Blood sampling facilities far from the laboratory may benefit from using these tubes, thus diminishing preanalytical errors or time consuming transferring of plasma samples to secondary tubes.
